# Limonin Inhibits IL-1*β*-Induced Inflammation and Catabolism in Chondrocytes and Ameliorates Osteoarthritis by Activating Nrf2

**DOI:** 10.1155/2021/7292512

**Published:** 2021-11-09

**Authors:** Jie Jin, Xinhuang Lv, Ben Wang, Chenghao Ren, Jingtao Jiang, Hongyu Chen, Ximiao Chen, Mingbao Gu, Zongyou Pan, Naifeng Tian, Aimin Wu, Liaojun Sun, Weiyang Gao, Xiangyang Wang, Xiaolei Zhang, Yaosen Wu, Yifei Zhou

**Affiliations:** ^1^Department of Orthopaedics, The Second Affiliated Hospital and Yuying Children's Hospital of Wenzhou Medical University, Wenzhou, Zhejiang Province, China; ^2^Zhejiang Provincial Key Laboratory of Orthopedics, Wenzhou, Zhejiang Province, China; ^3^The Second School of Medicine, Wenzhou Medical University, Wenzhou, Zhejiang Province, China; ^4^Research Institute of Experimental Neurobiology, Department of Neurology, The First Affiliated Hospital, Wenzhou Medical University, Wenzhou, Zhejiang Province, China; ^5^Chinese Orthopaedic Regenerative Medicine Society, Hangzhou, Zhejiang Province, China; ^6^Department of Orthopaedics, The Second Affiliated Hospital of Zhejiang University School of Medicine, Hangzhou, Zhejiang Province, China

## Abstract

Osteoarthritis (OA), a degenerative disorder, is considered to be one of the most common forms of arthritis. Limonin (Lim) is extracted from lemons and other citrus fruits. Limonin has been reported to have anti-inflammatory effects, while inflammation is a major cause of OA; thus, we propose that limonin may have a therapeutic effect on OA. In this study, the therapeutic effect of limonin on OA was assessed in chondrocytes *in vitro* in IL-1*β* induced OA and in the destabilization of the medial meniscus (DMM) mice *in vivo*. The Nrf2/HO-1/NF-*κ*B signaling pathway was evaluated to illustrate the working mechanism of limonin on OA in chondrocytes. In this study, it was found that limonin can reduce the level of IL-1*β* induced proinflammatory cytokines such as INOS, COX-2, PGE2, NO, TNF-*α*, and IL-6. Limonin can also diminish the biosynthesis of IL-1*β*-stimulated chondrogenic catabolic enzymes such as MMP13 and ADAMTS5 in chondrocytes. The research on the mechanism study demonstrated that limonin exerts its protective effect on OA through the Nrf2/HO-1/NF-*κ*B signaling pathway. Taken together, the present study shows that limonin may activate the Nrf2/HO-1/NF-*κ*B pathway to alleviate OA, making it a candidate therapeutic agent for OA.

## 1. Introduction

Osteoarthritis (OA), a common orthopedic disease, is also known as degenerative arthritis that primarily affects elderly people [1]. As reported in studies, the incidence of knee osteoarthritis is increasing at a high speed, causing a substantial economic burden to the family and the healthcare system [2, 3] . OA mainly affects the hip and knee joints with symptoms gradually worsening over time [4]. Synovial, cartilage, subchondral bone, joints pain, and limited movement are the most common symptoms of OA [5]. Various factors such as age, sex, trauma, obesity, and heredity have been implicated as contributing factors to OA [6, 7]. However, the exact pathogenesis of OA is remaining elusive [8]. Existing medications are symptomatic based, which provide temporary relief from the disease and are accompanied by many side effects [9]. With this consideration, it is imperative to identify new candidate drugs that can reverse OA with minimal side effects.

IL-1*β* is considered to be a major inducer of OA [10]. IL-1*β* could promote the cleavage of the cartilage matrix by activating the expression of matrix metalloproteinases (MMPs) and a disintegrin and metalloproteinase with thrombospondin motifs (ADAMTSs), thus leading to degradation of extracellular matrix (ECM), such as Collagen II and Aggrecan during OA pathogenesis [11]. Furthermore, the release of inflammatory mediators like prostaglandin E2 (PGE2) and nitric oxide (NO) may be triggered by IL-1*β* [12], while excessive inflammation and ECM degradation may significantly contribute to the pathogenesis of OA.

The nuclear factor kappa B (NF-*κ*B) signaling pathway is a downstream effector of IL-1*β* [13], it may stimulate inflammation and catabolism in chondrocytes. When IL-1*β* stimulates chondrocytes, the phosphorylated IкB*α* degrades in the cytoplasm, leading to the nuclear translocation of p65, which may further promote the expression of inflammatory and catabolic genes [14]. Studies have revealed that the activation of Nrf2/HO-1 signaling may suppress the NF-*κ*B pathway [15, 16]. Knockout of Nrf2 in mice showed more severe cartilage damage than wild-type mice [17], while activation of Nrf2 in chondrocytes may alleviate the pathology of OA [18], demonstrating that Nrf2 exhibits a protective role in cartilage. Therefore, the activation of Nrf2 probably serves as a potential therapy for OA.

Limonin, a highly oxidized compound, belongs to the triterpenoid class and is mainly extracted from tangerine fruits, including tangerines, oranges, and grapefruits, and it is widely used in dietary supplements [19, 20]. Limonin exhibits several significant biological and pharmaceutical activities, including antioxidant, antibacterial, antitumor, analgesic, and anti-inflammatory effects [21, 22]. Limonin has been reported to suppress the NF-*κ*B pathway in RAW 264.7 macrophages stimulated by LPS [23]. Meanwhile, it has also been shown that limonin may activate Nrf2 in HepG2 cells [24]. Although studies have described the regulatory effects of limonin on the NF-*κ*B pathway and Nrf2, the effect of limonin in OA is still unknown.

In this study, we evaluated whether limonin has an anti-inflammation and anti-ECM degradation potential in mouse chondrocytes *in vitro* and in DMM OA mice models *in vivo* and whether Nrf2/HO-1/NF-*κ*B cascades contribute to the effect of limonin on OA.

## 2. Materials and Methods

### 2.1. Reagents and Antibodies

Limonin (purity ≥ 98%), DMSO, Safranin O, collagenase type-II, Fast Green FCF, and hematoxylin were provided by Solarbio (Beijing, China). Recombinant rat IL-1*β* was purchased from Multi Sciences Biotech, Co., Ltd. (Hangzhou, China). Anti-Collagen II, Aggrecan, ADAMTS5, and MMP13 were purchased from Abcam (Cambridge, UK). Anti-p65, I*κ*B*α*, and Lamin-B1 were purchased from Cell Signaling Technology (Danvers, MA, USA). Anti-INOS, COX-2, HO-1, NQO1, SOD1, Nrf2, and GAPDH were purchased from Proteintech (Wuhan, China). Reagents for cell culturing were purchased from Gibco (Grand Island, USA).

### 2.2. Culturing of Primary Mouse Chondrocytes

Two-week-old mice (C57BL/6) were sacrificed with pentobarbital overdose. Under aseptic conditions, the articular cartilage of the knee joints was taken and minced with a sterile blade. After that, the articular cartilage was washed with PBS for three times and then exposed to collagenase type II (0.2%) in a 37°C incubator for 4 hrs. The digested cartilage tissue was centrifuged at 3000 rpm for 5 min. After the supernatant was extracted, the pellets in chondrocytes were resuspended in an incubator filled with DMEM/F12 and FBS (10%) and penicillin/streptomycin antibiotics (1%), and the incubator was stored in CO_2_ (5%) at 37°C. When chondrocytes reached 80% ~90% confluence, cells were collected by trypsin-EDTA (0.25%). Then, the cells were seeded in a 10 cm^2^ petri dish at an appropriate concentration (about 1 × 10^5^ cells/ml) 48 hrs later. To reduce experimental errors, we use second-generation chondrocytes.

### 2.3. Animal Model

A total of 60 wild-type (WT) C57BL/6 male mice of eight weeks were used for animal experiments. The *in vivo* studies were carried out according to the guidelines of the Animal Care and Use Committee of Wenzhou Medical University. As previously documented, an OA mouse model with unstable medial meniscus (DMM) was established surgically [25]. The operation procedure is as follows: all mice were anesthetized through an injection of 2% (*w*/*v*) pentobarbital sodium (40 mg/kg); next, the right knee joint capsule located in the tendon, bone, and medial meniscus tibial ligament was then cut open, and the medial meniscus tibial ligament was cut with microsurgical scissors, which were taken as the DMM group, while those whose medial meniscus tibial ligaments were not cut were taken as the sham group. Sixty C57BL/6 mice were fed adaptively for one week before operation. The treatment was carried out on the second day after operation. In the DMM+limonin group, limonin (40 mg/kg/d, soluble in saline) was injected intraperitoneally for 8 weeks. The sham+saline group and the DMM+saline group were separately injected with the same amount of saline. In addition, all mice were placed in an air-conditioned room (with a temperature of 23 ± 2°C, a humidity of 50 ± 10%, and a 12/12 h light/dark cycle), and they were given access to enough water and food. After 8 weeks, all mice were euthanized. Specimens (cartilage) were gathered for histological and X-ray examination.

### 2.4. Cell Viability Assay

According to the manufacturer's instructions, the CCK-8 kit was used to evaluate the effects of limonin on the activity of chondrocytes. Second-generation chondrocytes were seeded in 96-well plates (8000 cells/well) and exposed to limonin at different concentrations (0, 15, 30, 60, and 120 *μ*M) at 24 or 48 hrs. After that, each well was treated with 10 *μ*l CCK-8 and incubated for another 2 hrs at 37°C. Subsequently, the density of the O.D. was recorded by a spectrophotometer (Thermo Fisher) at 450 nm. All assays were performed in triplicate.

### 2.5. PGE2, NO, TNF-*α*, and IL-6 Measurements

Griess reaction was used to evaluate the NO interaction in the medium, as discussed earlier [26]. In the supernatant of the cell culture, different concentrations of PGE2, TNF-*α*, and IL-6 were evaluated by using commercial ELISA kits. The obtained results were repeated in triplicate.

### 2.6. Quantitative RT-PCR Experiments

The extraction of total RNA from chondrocytes was carried out *via* TRIzol reagent (Invitrogen) and stimulated by IL-1*β* (10 ng/ml) with different concentrations of limonin. Similarly, through reverse transcriptase, the cDNA was synthesized using total RNA (1000 ng). For quantitative RT-PCR (qPCR), 2× SYBR Master Mix (5 *μ*l), each primer (0.25 *μ*l), and diluted cDNA (4.5 *μ*l) were used, and the total reaction volume was 10 *μ*l. By collecting the circulating threshold (Ct) and normalizing it to GAPDH. The 2^-*ΔΔ*CT^ method was considered to measure relative mRNA levels of all target genes. Primers for COX-2, INOS, IL-6, and TNF-*α* were designed with the help of the NCBI Primer-Blast Tool: INOS: (F)5′-CGGAGAACAGCAGAGTTGG-3′ (R)5′-GGAATAGCACCTGGGGTTT-3′; COX-2: (F)5′-ACTCTATCACTGGCATCCG-3′ (R)5′-GAGCAAGTCCGTGTTCAAG-3′; TNF-*α*: (F)5′-CCACCACGCTCTTCTGTC-3′ (R)5′-GCTACGGGCTTGTCACTC-3′; and IL-6: (F)5′-CACCAGGAACGAAAGTCAA-3′(R)5′-CAACAACATCAGTCCCAAGA-3′ GAPDH:(F)5-′GTTGTGGCTCTGACATGCT-3′ (R) 5′-CCCAGGATGCCCTTTAGT-3′.

### 2.7. Western Blotting

The total protein isolation of chondrocytes was carried out with a RIPA lysis buffer comprising PMSF (phenylmethanesulfonylfluoride, 1 mM). Next, the mixture was centrifuged at 4°C for 15 min at 12000 rpm, and the lysates were placed for on ice 10 min. The protein concentrations were evaluated by the BCA protein assay kits (Beyotime). SDS PAGE was employed to separate proteins, and PVDF membranes were used to transfer 40 ng of protein, and then, the membranes were blocked by skimmed milk (5%) for 2 hrs. After that, the membranes were incubated with primary antibodies for 24 hrs at 4°C. The ratios of INOS, COX-2, Collagen II, ADAMTS5, p65, Nrf2, HO-1, I*κ*B*α*, and Lamin-B1 were the same ratio (1 : 1000), while the ratio of GAPDH was 1 : 3000. After that, TBST was used to wash the membrane. Then, the membrane was incubated with specific secondary antibodies for 2 hrs at 25°C. Electrochemiluminescence plus reagent was used to visualize the blots. After being washed with TBST for three times. Eventually, the intensity of underlined blots was visualized by the Bio-Rad Imaging System.

### 2.8. siRNA Transfection

The specific Nrf2 siRNA and HO-1 siRNA were purchased from Guangzhou RiboBio Co. Ltd. The sequences of Nrf2 siRNA were as follows: sense, 5′-CAAACAGAATGGACCTAAA-3′. The sequences of HO-1 siRNA were as follows: sense, 5′-CAAGGAGGTACACATCCAA-3′. Nrf2, HO-1, and negative control were used to transfect siRNA according to steps of the manufacturer.

### 2.9. Immunofluorescence

Second-generation chondrocytes were seeded on 6-well plates, by using coverslips, and the cells were exposed to IL-1*β* (10 ng/ml) or cotreated with 10 ng/ml of IL-1*β* and limonin at a concentration of 60 *μ*M for in the medium overnight after incubation with a serum-deprived medium for 24 hrs. Next, the coverslips with monolayers of the chondrocytes were washed with PBS for three times, and the cells were fixed with paraformaldehyde (4%) at ~25°C for 15 min and then rinsed with PBS again. Triton X-100 (0.1%) was for the cells and nuclear membrane permeability for 5 min at 25°C. Next, the cells were blocked with BSA (5%) at 37°C for 60 min, washed with PBS, and incubated with primary antibodies: Collagen II (1: 200), MMP13 (1 : 200), p65 (1: 200), and Nrf2 (1 : 200) at 4°C for 24 hrs. And then, the cells were incubated with secondary antibodies (1 : 400) at 25°C for 60 min after being washed with PBS. After that, this was followed by the cells being labeled with DAPI (Invitrogen) for 1 min. The slides were imaged by the Olympus laser scanning microscope, and the fluorescence intensity was evaluated by ImageJ (Bethesda, MD, USA).

### 2.10. Immunohistochemistry (IHC) and Tissue Fluorescence

The paraffin sections of the knee joint were deparaffinized by xylene and rehydrated in ethanol. The sections were washed with PBS, blocked with 3% H_2_O_2_, and then placed in 95°C sodium citrate buffer for 10 min. Later, they were blocked with 10% bovine serum albumin phosphate-buffered saline for 10 min. Finally, the sections were incubated at 4°C overnight with the following primary antibodies: Nrf2 (1 : 200), HO-1 (1 : 200), p65 (1 : 200), ADAMTS5 (1 : 200), MMP13 (1 : 200), TNF-*α* (1 : 200), IL-6 (1 : 200), and NQO1 (1 : 200). The next day, the sections were incubated with a secondary antibody conjugated to horseradish peroxidase and counterstained with hematoxylin. Then, the nuclei were stained with DAPI. The image was taken by an optical microscope. For histofluorescence, the previous steps were the same as IHC, and then, the samples were incubated with primary antibodies against INOS (1 : 200) and SOD1 (1 : 200) at 4°C overnight. The samples were then incubated with secondary antibodies at room temperature for 1 h, and the nucleus was stained with DAPI. The slides were imaged by an Olympus laser scanning microscope, and the fluorescence intensity was evaluated *via* ImageJ software 2.1 (Bethesda, MD, USA).

### 2.11. Molecular Modeling

The structure data file (SDF) of the compound limonin was retrieved from the PubChem website, and the recipient was retrieved from the Protein Data Bank database. For somatic protein Nrf2 (PDB ID: 4ZY3), PYMOL 2.3.4 software was adopted to perform such operations as water removal and ligand removal on the receptor protein, AutoDockTools software was used to modify the receptor protein such as hydrogenation and charge balance, and AutoDock Vina 1.1. 2 was used to perform molecular docking of receptor protein and ligand small molecule. Eventually, the affinity was evaluated.

### 2.12. X-Ray Imaging Method

Eight weeks after surgery, an X-ray was carried out on all mice with a digital X-ray machine to evaluate variations in joint space, osteophyte development, and calcification on the surface of the cartilage. Accurate images were obtained through underlined settings: 50 kv and 160 *μ*A.

### 2.13. Histopathological Evaluations

All joints slices were stained with saffron O-fast green (S-O). The cellular morphology of cartilages and subchondral bones were randomly evaluated by a microscope *via* the OARSI system through three-level sections of the joint, containing the medial femoral condyle and medial plateau. 15 mice were used in each group.

### 2.14. Statistical Analysis

The assays were carried out for three times, and statistical analysis was performed by SPSS 20.0. The results were tested by ANOVA, and then, Tukey's test was done to compare the difference between the control and treatment groups. Nonparametric data, including the OARSI score, was evaluated by Kruskal-Wallis *H*, and *p* < 0.05 was considered to be significant.

## 3. Results

### 3.1. Effects of Limonin on the Survival of Chondrocytes

**Figure 1:**
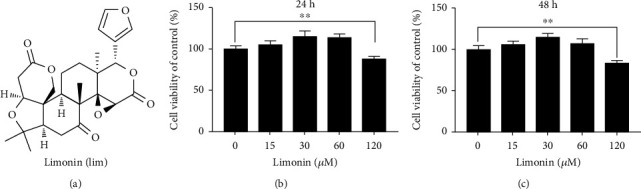
The effect of limonin on the survival of chondrocytes *in vitro*. (a) The chemical structure of limonin. (b, c) The toxic effect of limonin on chondrocytes at different concentrations (0, 15, 30, 60, and 120 *μ*M) for 24 and 48 hrs. The viability of cells was assessed by CCK-8 assay. The results of three independent experiments are represented as mean ± S.D. Significant differences are represented as ^∗∗^*p* < 0.01, *n* = 3.

The chemical structure of limonin is shown in Figure 1(a). To estimate the cytotoxic effect of limonin, chondrocytes were exposed to limonin at different concentrations (0, 15, 30, 60, and 120 *μ*M) for 24 or 48 hrs, and the survival of cells was evaluated by CCK-8 assay. As shown in Figures 1(b) and 1(c), after 48 hrs of treatment, limonin reduced the viability of chondrocytes at 120 *μ*M, suggesting that limonin showed no cytotoxicity to chondrocytes after 24 or 48 hrs below 60 *μ*M concentrations. Therefore, different concentrations (15, 30, and 60 *μ*M) of limonin were used in the study.

### 3.2. Limonin Attenuates IL-1*β*-Activated INOS, COX-2, PGE2, NO, IL-6, and TNF-*α* Expressions in Chondrocytes

**Figure 2:**
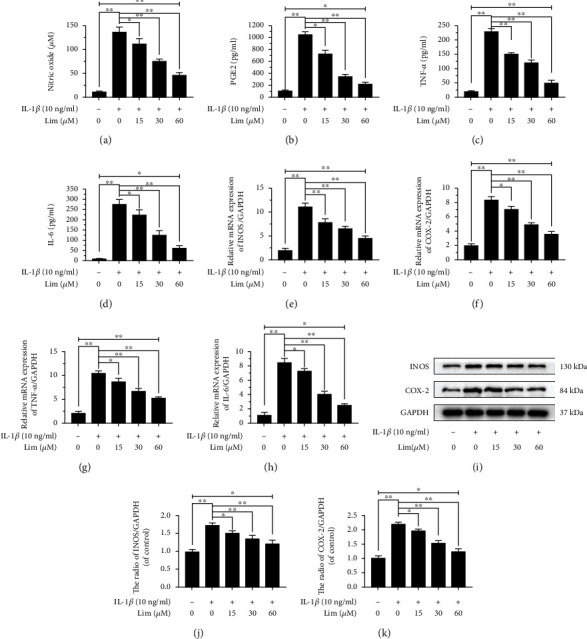
The effect of limonin on IL-1*β*-stimulated NO, PGE2, TNF-*α*, and IL-6 synthesis in chondrocytes. The pretreatment of chondrocytes with limonin at different concentrations (0, 15, 30, and 60 *μ*M) for 24 hrs. (a) In the culture medium, the levels of NO were evaluated by Griess reaction. (b–d) The concentrations of PGE2, TNF-*α*, and IL-6 were evaluated by ELISAs. (e–h) The levels of mRNA expression of INOS, COX-2, TNF-*α*, and IL-6 were evaluated by RT-PCR. (i-k) The protein expression of COX-2 and INOS was evaluated by western blot assay. The results are expressed as the averages ± S.D. Significant differences among various groups are represented as ^∗^*p* < 0.05, ^∗∗^*p* < 0.01; *n* = 3.

In this paper, we determined the effect of limonin on IL-1*β*-activated inflammatory activity in chondrocytes. As shown in Figures 2(a)–2(d), the concentrations of NO, PGE2, TNF-*α*, and IL-6 were decreased gradually in the limonin treatment group but still were higher than that of the control group and lower than that of the IL-1*β* group. In addition, according to the results of western blot assay and RT-PCR, the mRNA and protein expression levels of INOS and COX-2 and the mRNA expression levels of IL-6 and TNF-*α* were decreased gradually in the limonin treatment group but were still higher than that of the control group and lower than that of IL-1*β* group (Figures 2(e)–2(k)). Together, these results meant that limonin partially reversed IL-1*β*-mediated inflammation.

### 3.3. Effects of Limonin on the Metabolism of ECM in Chondrocytes

**Figure 3:**
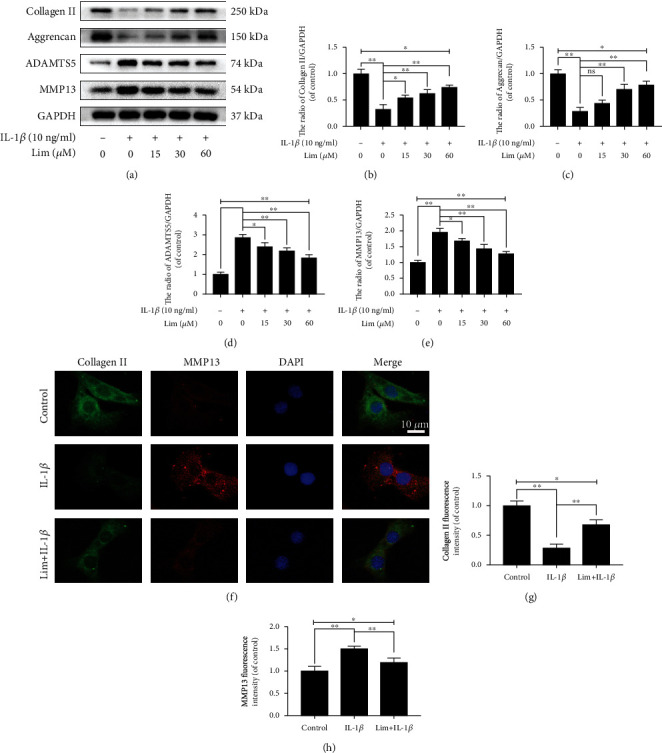
The effect of limonin on IL-1*β*-activated ECM degradation in chondrocytes. (a–e) The protein expression of Aggrecan, Collagen II, ADAMTS5, and MMP13 in chondrocytes was evaluated by western blot assay. (f–h) The protein expression of Collagen II and MMP13 was evaluated by cell immunofluorescence (scale bar: 10 *μ*m) and ImageJ. The results in the figures are represented as averages ± S.D. Significant differences among various groups are represented as ^∗^*p* < 0.05, ^∗∗^*p* < 0.01; *n* = 3.

To determine the role of limonin in IL-1*β*-stimulated ECM degradation, the protein expression of Aggrecan, Collagen II, ADAMTS5, and MMP13 was detected by western blotting. As shown in Figures 3(a)–3(e), compared with the IL-1*β* group, the protein expression of Collagen II and Aggrecan was increased gradually and the protein expression of ADAMTS5 and MMP13 was decreased gradually in the limonin treatment group. But, the protein expression of Collagen II and Aggrecan lowered than that of the control group, and the protein expression of ADAMTS5 and MMP13 was higher than that of the control group. In conclusion, these results meant that limonin partially reversed IL-1*β*-mediated ECM degradation.

In the following part, we assessed that the fluorescence intensity of Collagen II was decreased upon the administration of IL-1*β*, indicating that Collagen II expression was downregulated, while the fluorescence intensity of MMP13 was increased, showing that the expression of MMP13 was enhanced. But, in the limonin treatment group, Collagen II fluorescence intensity increased, while MMP13 fluorescence intensity decreased, indicating that limonin can inhibit ECM degradation (Figures 3(f)–3(h)). Since MMP13 can specifically degradate Collagen II, thus, the immunofluorescence assays of MMP13 and Collagen II may not only validate the expression results by western blot assay but also show the dynamic degradation process of Collagen II by MMP13 *via* colocalization. As we could see from the figure that when MMP13 is lowly expressed, the content of Collagen II was high (control group), however, when MMP13 was highly expressed, the content of Collagen II was low (IL-1*β* group). In short, limonin could effectively inhibit the effect of IL-1*β* on ECM degradation.

### 3.4. Limonin Inhibits the NF-*κ*B Signaling Activated by IL-1*β*

**Figure 4:**
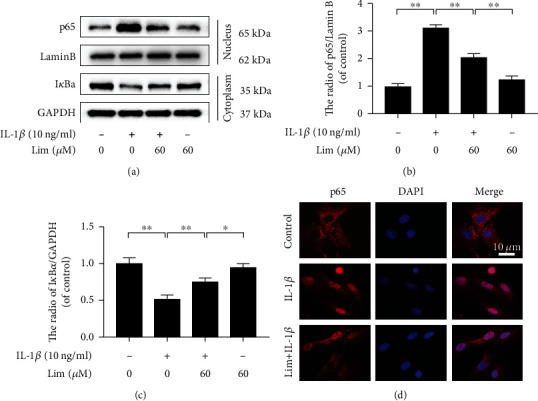
The effect of limonin on IL-1*β*-activated NF-*κ*B stimulation. (a–c) The western blot assay was used to evaluate the I*κ*B*α* protein expression in the cytoplasm and p65 expression in the nucleus in chondrocytes. (d) The p65 in the nuclear was assayed through the cell immunofluorescence (scale bar: 10 *μ*m). The results in the figures are represented as the averages ± S.D. Significant differences among various groups are shown as ^∗∗^*p* < 0.01, *n* = 3.

To better understand the potential mechanisms of the anti-inflammatory and anti-ECM degradation effect of limonin, the influence of the NF-*κ*B cascade on chondrocytes was evaluated. The expression of cytoplasmic protein I*κ*B*α* and nuclear protein p65 in mouse chondrocytes was evaluated by western blot assay. When activated by IL-1*β*, p65 was observed to increase in the nucleus, while the cytoplasmic degradation of I*κ*B*α* increased (Figures 4(a)–4(c)). At the same time, the immunofluorescence staining data showed that the IL-1*β*-promoted translocation of p65 from the cytoplasm to the nucleus was significantly inhibited by limonin (Figure 4(d)).

### 3.5. Limonin Activates Nrf2/HO-1 Cascade in Chondrocytes

**Figure 5:**
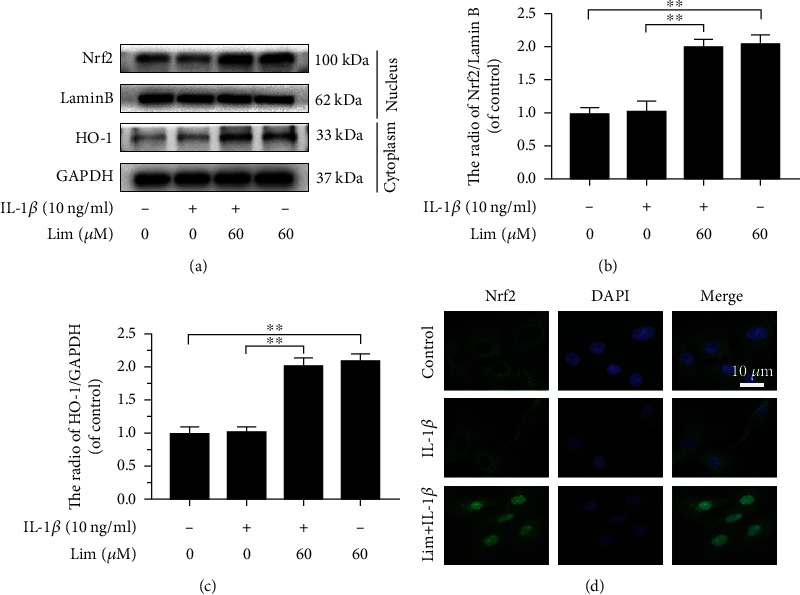
The effect of limonin on the Nrf2/HO-1 cascade. (a–c) The protein expression of Nrf2 and HO-1 was evaluated by western blot assay. (d) The Nrf2 in the nuclear was assayed through cell immunofluorescence (scale bar: 10 *μ*m). The results are represented as the mean ± S.D. Significant differences among various groups are represented as ^∗∗^*p* < 0.01, *n* = 3.

Even though earlier a research report has revealed that limonin activates the Nrf2/HO-1 cascade [24], its role in chondrocytes is not yet known. The western blot assay results showed that limonin enhanced the transportation of Nrf2 into the nucleus and the expression of HO-1 in the cytoplasm, as shown in Figures 5(a)–5(c). Simultaneously, IL-1*β* activation did not influence the expression level of HO-1 and Nrf2. The results of immunofluorescence staining revealed that limonin enhanced the fluorescence and the nuclear intensity of Nrf2 in IL-1*β* activated chondrocytes (Figure 5(d)), and these results showed consistency with the results of immunoblotting. In a word, limonin activates Nrf2/HO-1 cascade in chondrocytes.

### 3.6. Molecular Docking between Limonin and Nrf2

**Figure 6:**
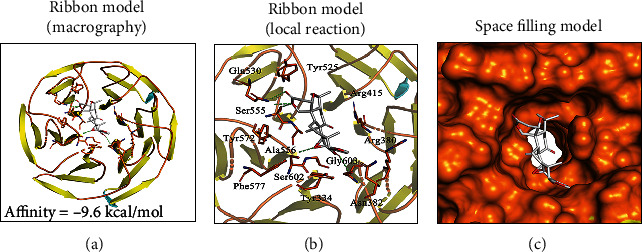
Limonin interacts with Nrf2 in a docking study. A ribbon model is used to demonstrate the binding mode between the receptor protein Nrf2 and limonin ligand small molecules. (a) Limonin was able to dock strongly within the Nrf2 binding site (affinity = −9.6 kcal/mol). (b) This proposed binding interacts with Ser555, Gly603, and Ala556 on Nrf2. (c) The binding of the Nrf2 pocket is shown with a space-filling model.

We next evaluated whether there is any affinity between limonin and Nrf2 protein by computational molecular docking analysis [27]. In this analysis, we employed the chemical structure of limonin, as shown in Figure 1(a). After examining all the generated models, we discovered that limonin interacts with and docks at the Nrf2 binding site (Figure 6), and the macro- and local-level views of these interactions were displayed with a ribbon model (Figures 6(a) and 6(b)). We also used a space-filling model to illustrate this kind of interaction (Figure 6(c)). High-affinity (-9.6 kcal/mol) hydrogen binding events were observed between the residues of Ser555, Gly603, and Ala556 in limonin and Nrf2. In conclusion, these results indicated that limonin probably inhibited the development of OA by interacting with the Nrf2 and promoting nuclear translocation.

### 3.7. Limonin Suppresses Inflammation and ECM Degradation in Chondrocytes via Nrf2

**Figure 7:**
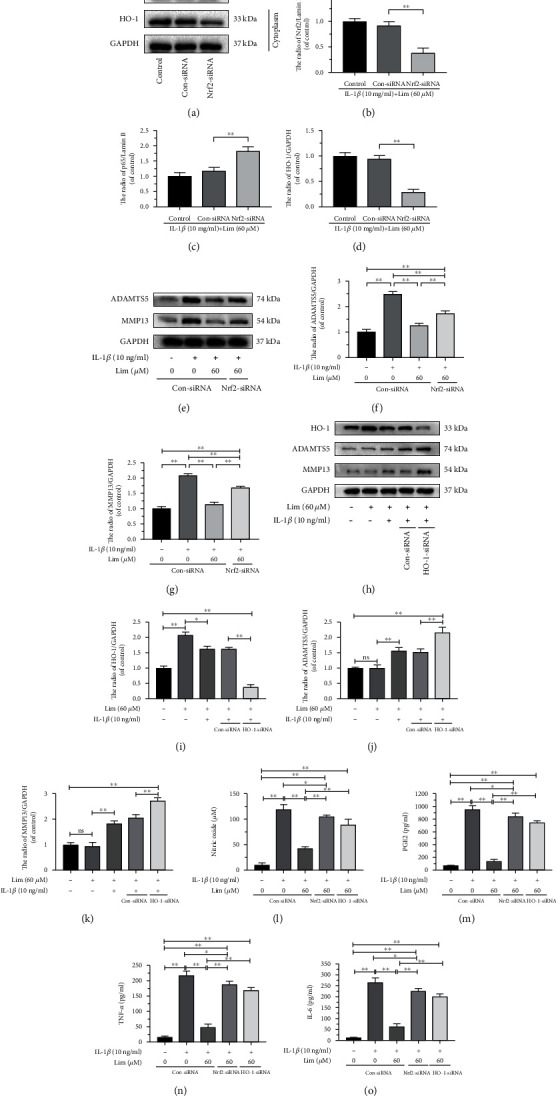
The protective effects of limonin in IL-1*β*-activated chondrocytes were reversed by Nrf2-siRNA and HO-1-siRNA. After Nrf2 was knocked down. (a–d) The protein expression of Nrf2, HO-1, and p65 was evaluated by western blot assay. (e–g) The protein expression of ADAMTS5 and MMP13 was evaluated by western blot assay. (h–k) After HO-1 was knocked down, the protein expression of HO-1, MMP13, and ADAMTS5 was evaluated by western blot assay. (l–o) The synthesis of NO, PGE2, TNF-*α*, and IL-6 in chondrocytes was evaluated by Griess reaction or ELISA. All results are represented as the mean ± S.D. Significant differences among various groups are represented as ^∗^*p* < 0.05, ^∗∗^*p* < 0.01; *n* = 3.

To evaluate the contribution of Nrf2 in the limonin-triggered protective effects, Nrf2-siRNA was transfected into chondrocytes before limonin exposure. The transfection of Nrf2 siRNA attenuated the signaling cascade of Nrf2/HO-1 that was activated by limonin under the stimulation of IL-1*β*. In addition, after Nrf2 siRNA was transfected, the p65 expression was elevated in chondrocytes cotreated with limonin and IL-1*β*, which revealed that the Nrf2/HO-1 cascade facilitated the NF-*κ*B signaling inhibited by limonin (Figures 7(a)–7(d)). Moreover, western blot assay or Griess reaction or ELISA were employed to evaluate the expression of MMP13, ADAMTS5, PGE2, NO, TNF-*α*, and IL-6. As shown in Figures 7(e)–7(g) and Figures 7(l)–7(o), after the knockdown of Nrf2, ECM degradation and the expression of inflammation were increased, but still, they were lower than that of the IL-1*β* group and higher than that of the control group, which meant that knockdown of Nrf2 partially reduced the inhibitory effect of limonin on IL-1*β*-induced ECM degradation and inflammation. Therefore, our study confirmed that the Nrf2 pathway was necessary, not sufficient, for limonin.

To determine the role of HO-1, the protein expression of MMP13, ADAMTS5, NO, PGE2, TNF-*α*, and IL-6 was detected by western blot assay or Griess reaction or ELISA, by using HO-1 siRNA. The results showed that HO-1 expression increased with limonin treatment, and after administration of IL-1*β*, HO-1 siRNA significantly inhibited the therapeutic effect of limonin, making ECM degradation and inflammation increased, as shown in Figures 7(h)–(o). Accordingly, we may conclude that limonin can reduce inflammation and ECM degradation through thr Nrf2/HO-1 pathway.

### 3.8. Limonin Attenuates OA Progression in Mice

**Figure 8:**
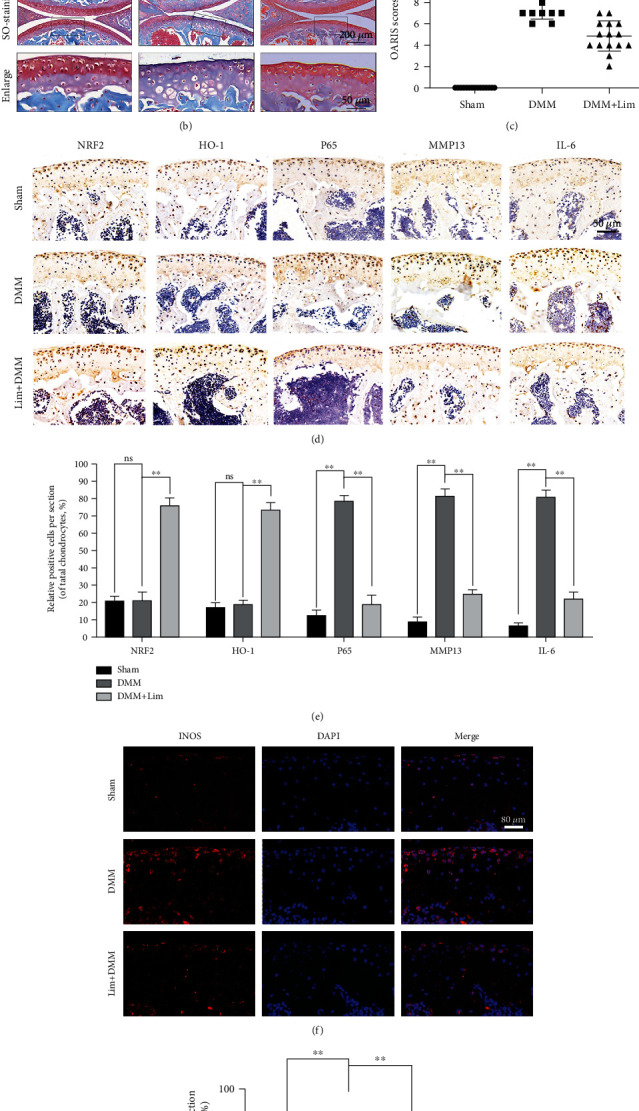
Amelioration of OA development in the DMM mouse model *in vivo* by limonin. (a) Images of mouse knee joints from various experimental groups were taken by digital X-ray. White arrows indicated the calcification of the cartilage surface. (b) Safranin O staining was used to analyze the morphometric differences among the sham group, the DMM group, and the limonin treatment groups (scale bar: 200 *μ*m or 50 *μ*m). (c) OARIS scores of all cartilages are indicated in diagrams (*n* = 15). (d) IHC of Nrf2, HO-1, p65, MMP13, and IL-6 was employed to evaluate the effect of limonin on the cartilage in OA models (scale bar: 50 *μ*m). (e) Quantitative analysis of positive cells in cartilage. (f, g) The protein expression of INOS in the OA model. All results are represented as the mean ± S.D. Significant differences among various groups are represented as ^∗∗^*p* < 0.01.

To validate the protective effect of limonin on OA, a DMM model was set up in mice and limonin was injected for 8 weeks. According to the X-ray results, cartilage calcification was not serious in the limonin treatment group (Figure 8(a)). The SO-staining results showed that the joint surface of the DMM group was severely eroded, with less proteoglycan and fewer cells. However, in the limonin treatment group, the erosion was mild, and proteoglycan was higher than that of the DMM group (Figure 8(b)). Meanwhile, the OARSI score was lowered after limonin treatment than that of the DMM group, which was consistent with SO staining (Figure 8(c)).

The histochemical results of Nrf2, HO-1, p65, MMP13, ADAMTS5, IL-6, and TNF-*α* showed that the protein expression of Nrf2, and HO-1 did not differ significantly between the sham group and the DMM group, while p65, MMP13, ADAMTS5, IL-6, and TNF-*α* changed significantly in the DMM group. However, in the limonin treatment group, the protein expression of Nrf2 and HO-1 was markedly increased, while the protein expression of p65, MMP13, IL-6 (Figures 8(d) and 8(e)), TNF-*α*, and ADAMTS5 (Figure S2(a)) was decreased. Moreover, as shown in Figures 8(f) and 8(g), the protein expression of INOS in the limonin treatment group was significantly lower than that of the DMM group. Together, these results suggested that limonin suppressed OA development in mice.

## 4. Discussion

Studies have been shown that inflammation and ECM degradation significantly contributes to OA development [28–30]. Currently, the commonly prescribed drugs for the treatment of OA are NSAIDs. Even if the drugs are mostly symptomatic-based that can delay OA symptoms, they cannot control OA development and have many side effects [31]. So, it is necessary to determine candidate drugs for the treatment of OA. In this study, we discovered that limonin inhibited IL-1*β*-induced inflammation and ECM degradation in chondrocytes. We further demonstrated that limonin improved the pathogenesis of OA *in vivo* and *in vitro* through the Nrf2/HO-1/NF-*κ*B pathway.

According to reported studies [32, 33], NF-*κ*B signaling significantly contributes to the development of OA. During the IL-1*β*-activated inflammatory response, I*κ*B kinase is activated and results in I*κ*B*α* degradation. After that, p65 translocates into the nucleus [34]. In the nucleus, p65 promotes the mRNA level of pro-inflammatory factors, including INOS, PGE2, TNF-*α*, COX-2, and IL-6 [14]; thus, it can aggravate OA. Furthermore, the effect of limonin on inflammation and ECM degradation is correlated with the attenuation of the NF-*κ*B signaling cascade. Hence, it is important to find a therapeutic target for inhibiting the NF-*κ*B signaling pathway. Some studies [35, 36] have reported that Nrf2 activation can promote the downstream gene HO-1 expression and inhibit the entry of p65 into the nucleus to reduce inflammation and ECM degradation, while the lack of Nrf2 can increase susceptibility to inflammatory disorders [37]. Therefore, we hypothesized that Nrf2 activated by limonin might play a protective role in OA.

In this study, as shown in Figure 5(a) and molecular docking (Figure 6), limonin activated Nrf2, increased HO-1 expression, and inhibited p65 expression. Next, to determine whether Nrf2 is necessary for the effect of limonin on chondrocytes, we used Nrf2-siRNA and we knocked down Nrf2; our results were similar to Luo et al. [38], demonstrating that Nrf2 could activate the downstream gene HO-1 and inhibit the p65 activation to reduce inflammation and ECM degradation. In the current study, the results revealed that Nrf2/HO-1 played an important role in anti-inflammation and anti-ECM degradation. However, increased Nrf2 translocation to the nucleus not only raised HO-1 protein levels but also several antioxidant enzymes. To determine the necessity of the role of HO-1, we also knocked down HO-1 and the results were similar to that of knocking down Nrf2, which further proved that limonin plays a role through Nrf2/HO-1 pathways.

In addition, considering that there is also redox in the OA model, we evaluated the redox state in the OA model. We detected the antioxidant proteins NQO1 and SOD1 in the OA model. The antioxidant protein NQO1 (Figure S2(a)) and SOD1 (Figure S2(c)) increased in the limonin treatment group. Besides, the Nrf2 and HO-1 were also important redox factors. Taking these factors into account, limonin may reduce the level of oxidative stress in osteoarthritis.

In order to determine the most effective therapeutic dose of limonin in mice, the DMM model was established to determine the effective therapeutic dose of limonin in mice. Before starting the experiment, we set a dose gradient of 20 mg/kg and 40 mg/kg for an 8-week pre-experiment. Limonin's therapeutic efficacy was assessed after 8 weeks of SO staining and OARSI scores. As shown in Figure S1(a)-S1(b), compared with the therapeutic dose of 20 mg/kg, the therapeutic dose of 40 mg/kg has a lower score and a more complete cartilage surface. Therefore, we chose a dose of 40 mg/kg throughout the experiment.

Furthermore, we also investigated whether limonin alleviates osteoarthritis in mice through any other pathways. Previous studies have reported that IL-1*β* can activate Myd88 [39, 40]. Myd88 is the most critical effector molecule in the signal transduction of TLRs. When TLRs recognize ligands, it may activate the downstream signaling through MyD88 and finally activate the nuclear factor *κ*B (NF-*κ*B) to regulate the expression of corresponding inflammatory cytokines [41, 42]. In addition, studies have shown that Nrf2 may regulate the TLR-4/MyD88/NF-*κ*B signaling pathway in specific pathological processes [43]. Therefore, limonin may exert its effect through Nrf2/MyD88/NF-*κ*B signaling pathway. Some studies also showed that IL-1*β* can activate MAPK and inflammasomes [44, 45], two major ways to induce inflammation in cells. Meanwhile, Nrf2 may also demonstrate to regulate MAPK and inflammasomes [46]. Thus, it is suggested that limonin may also exert its effect through MAPK and inflammasomes regulation, which certainly remains to be verified by further studies.

Moreover, there are several limitations to our study. Firstly, further *in vivo* characterization and functional studies are needed to demonstrate the clinical efficacy of limonin in OA. What is more, the effects of limonin should be tested in Nrf2-knockout mice. Our study has shown that limonin, one of the active ingredients of lemon, inhibited inflammation in chondrocytes and promoted extracellular matrix homeostasis. These results do not suggest a high dietary intake of lemons can replace smaller doses of purified limonin. Therefore, this is yet to be determined in future studies.

In summary, we found that limonin inhibits NF-*κ*B by activating the Nrf2/HO-1 cascade in chondrocytes, thus greatly reducing the inflammatory response and catabolism induced by IL-1*β*. Besides, the *in vivo* results were consistent with the *in vitro* experimental results. An *in vivo* model showed that limonin can enhance Nrf2 and HO-1 expressions and reversed ECM degradation and inflammation. In a word, our study shows that limonin can be used as a drug candidate for the treatment of OA.

## 5. Conclusion

**Figure 9:**
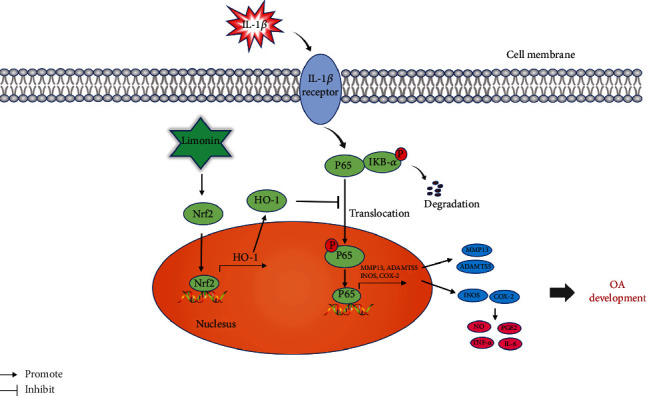
The limonin molecular mechanism contributes to the progression of OA.

Our study shows that limonin attenuates IL-1*β*-activated inflammation and ECM degradation by activating the Nrf2/HO-1 cascade and inhibiting NF-*κ*B; thus it protects OA (Figure 9).

## Data Availability

The data used to support the findings of this study are available from the corresponding authors upon request.

## Supplementary Materials

Supplementary Materials Figure S1: therapeutic effect of different doses of limonin on an OA model. All results are represented as the mean ± S.D. ^∗∗^*p* < 0.01. Figure S2: limonin increased the levels of antioxidant protein and diminished inflammation and ECM degradation in OA models. All results are represented as the mean ± S.D. ^∗∗^*p* < 0.01, *n* = 3.
