# Supplementary material for: Limonin Inhibits IL-1β-Induced Inflammation and Catabolism in Chondrocytes and Ameliorates Osteoarthritis by Activating Nrf2
Source: Oxid Med Cell Longev. 2021 Nov 9;2021:7292512. doi: 10.1155/2021/7292512 (PMC8595032; doi:10.1155/2021/7292512)
Supplement: Supplementary Materials — Figure S1: therapeutic effect of different doses of limonin on an OA model. All results are represented as the mean ± S.D. ∗∗p < 0.01. Figure S2: limonin increased the levels of antioxidant protein and diminished inflammation and ECM degradation in OA models. All results are represented as the mean ± S.D. ∗∗p < 0.01, n = 3. [file 7292512.f1.docx]

**Supplemetary material：**


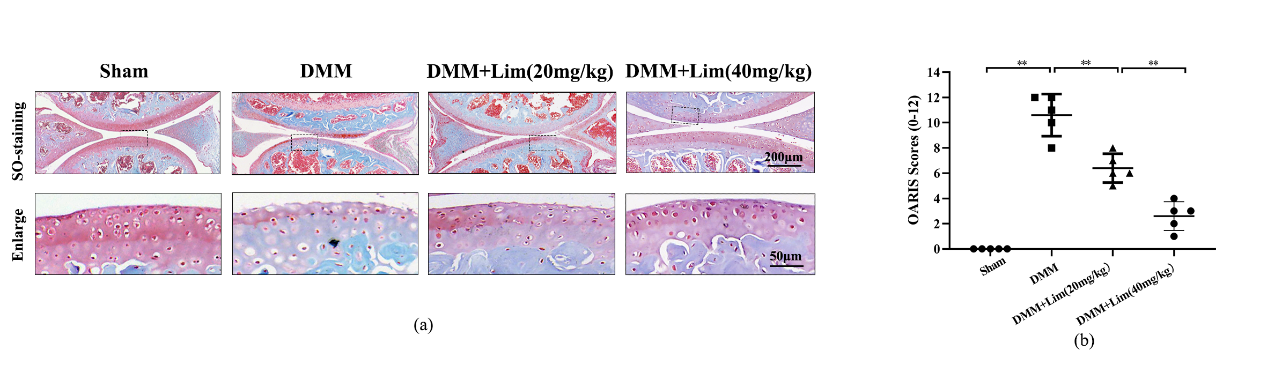


Figure S1: Therapeutic effect of different doses of limonin on OA model. (a) Safranin O staining was employed to analyze his morphometric variations among the sham + saline gruop, the DMM + saline gruop, the DMM+ limonin (20mg/kg) group, and the DMM+ limonin (40mg/kg) group (scale bar: 200 μm or 50 μm) (b) OARIS scores of all cartilages are indicated in diagrams (n=5). All results are represented as the mean ± S.D. Significant differences among various groups are represented as ***p* <0.01.


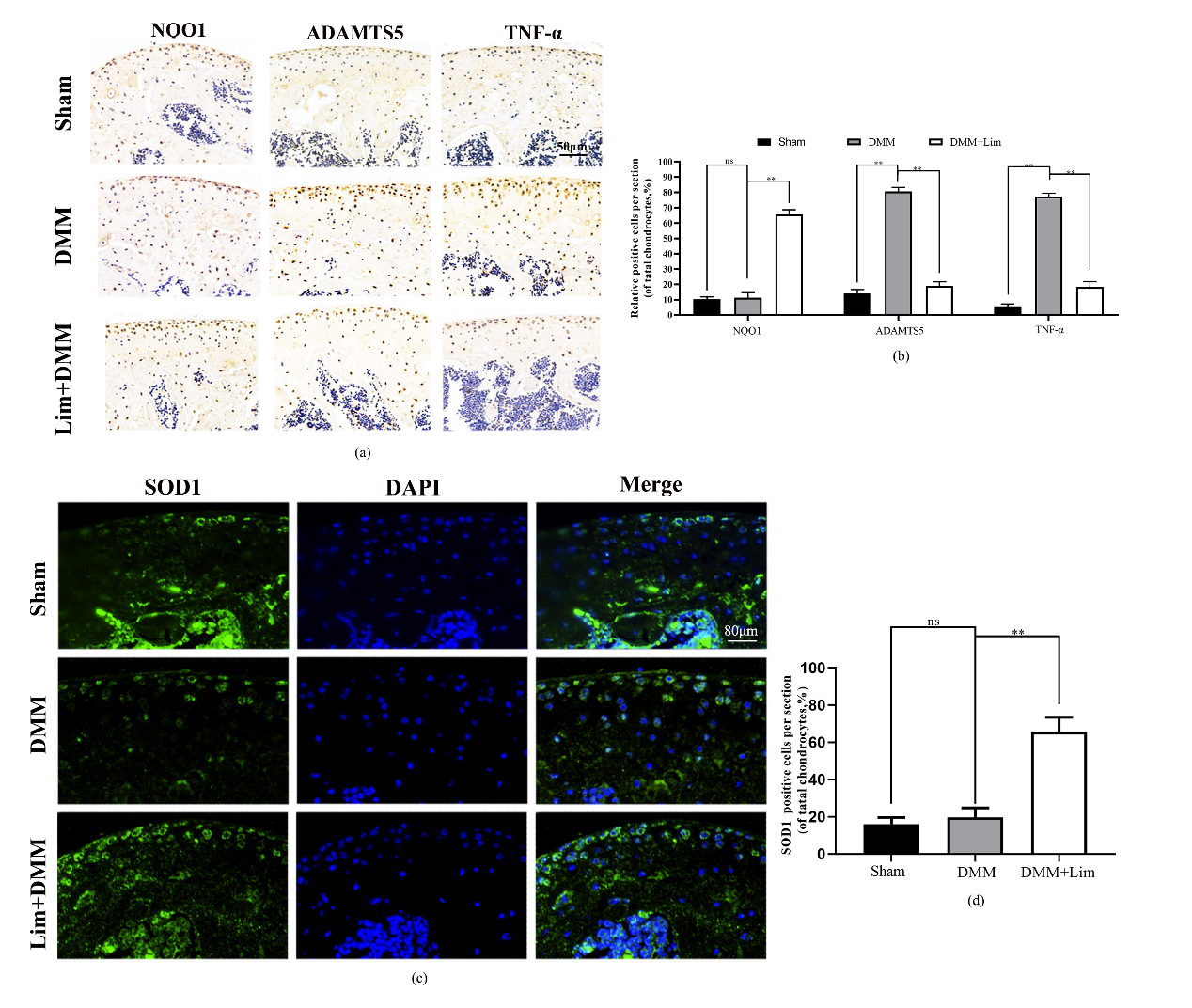


Figure S2: Limonin increased the levels of antioxidant protein, and diminished inflammation and ECM degradation in OA models. (a,b) The protein expression of NQO1, ADAMTS5, and TNF-α levels was detected in the OA model. (c,d) The protein expression of SOD1 was detected by tissue immunofluorescence. All results are represented as the mean ± S.D. Significant differences among various groups are represented as ***p*< 0.01, n =3
